# Idiopathic Low-Flow Priapism in Prepuberty: A Case Report and a Review of Literature

**DOI:** 10.1155/2008/549861

**Published:** 2008-09-29

**Authors:** Ihab A. Hekal, Eric J. H. Meuleman

**Affiliations:** ^1^Urology Department, Urology and Nephrology Center, Mansoura 35516, Egypt; ^2^Urology Department, Free University Medical Center, 1007 MB Amsterdam, The Netherlands

## Abstract

*Introduction*. The incidence of priapism in adults is higher than in children. Although approximately 50% of all episodes of priapism are thought to be idiopathic, there are a number of known specific causes of this disorder. In adults intracavernous therapy with papaverine, phentolamine, alprostadil or combinations of these agents is the most common cause of ischemic priapism. In children the most common etiology is sickle cell anemia for low-flow priapism or post-traumatic high-flow priapism. We present a 13-year-old boy, not sexually active presented to our outpatient clinic suffering from long standing (3.5 hours) sustained painful erection. To the best of our knowledge the idiopathic low-flow priapism in pre-pubertal boy was not reported before in literature. Our case is the first case to be reported in pre-pubertal age. *Conclusion*. In pre-pubertal boys idiopathic recurrent priapism is a rare condition. In the literature, several empirical therapies are described. Recently, it is postulated that a low dose of a PDE5 inhibitor. The early conservative management is the best treatment option to safe the corporeal smooth muscles from irreversible damage.

## 1. INTRODUCTION

Priapism is defined as involuntary prolonged penile erection caused by factors other than sexual arousal. It is classified into low flow (ischemic or venous) or high flow (nonischemic or arterial).

The incidence of priapism in adults is higher than in children. Although approximately 50% of all episodes of priapism are thought to be idiopathic, there are a number of known specific causes of this disorder. In adults, intracavernous therapy with papaverine, phentolamine, alprostadil, or combinations of these agents is the most common cause of ischemic priapism [1]. In children, the most common etiology is sickle cell anemia for low-flow priapism or posttraumatic high-flow priapism [2].

To the best of our knowledge, the idiopathic low-flow priapism in prepubertal boy was not reported before in literature. Our case is the first case to be reported in prepubertal age.

## 2. CASE REPORT

A 13-year-old boy, not sexually active, was presented to our outpatient clinic suffering from long-standing (3.5 hours) sustained painful erection. This condition was not preceded by trauma nor drug intake. He had a history of three similar attacks in the past 3 months that were managed by cavernosal aspiration in another hospital. On examination, the penis was erected with a soft glans. A trial with intracavernosal aspiration under sedation and analysis of the aspirated blood for oxygen saturation and PH was done: PH: 7:01, PCo2: 11.6 (arterial ref.: 4.5–6.1 kPa), PO2: 3 (arterial ref.: 10–13.5 kPa).

Laboratory investigation to rule out a haematological disorder revealed normal blood picture (hemoglobin: 8.1 m·mol/L (ref.: 7.4–10.3 m·mol/L), RBCs: 4.62 × 10^12^/L (ref.: 4.1–5.6 × 10^12^/L), WBCs: 5.6 × 10^9^/L (ref.: 4.3–13.5 × 10^9^/L), and platelets: 195 × 10^9^/L (ref.: 150–450 × 10^9^/L). Hemoglobin-S was negative. Although, based on these results, a high-flow priapism was unlikely, the presence of an arteriovenous fistula was excluded by penile and perineal duplex ultrasound.

Following cavernosal aspiration under sedation, the penis became flaccid and the boy was discharged from admission. Physical exercise and oral-low dose of acetylsalicylate (40 mg) and ice packs were recommended in case the priapism would recur at home.

One week later, the boy came for a followup visit. On examination, his penis was flaccid with an ecchymotic patch at the aspiration puncture site. He reported to have morning erections. He was advised to continue the conservative management with low dose of acetylsalicylate (40 mg).

## 3. DISCUSSION

Priapism is a condition first described by Tripe in 1845 [3]. It has been defined as a pathological condition of penile erection that persists beyond or is unrelated to sexual stimulation [4].

In children and prepubertal boys as well as in adults, it can be classified according to the aetiology into low-flow (ischemic) and high-flow priapism. Most common causes of the former are the use of medications and hematological conditions. Several medications have been implicated in ischemic priapism. Most notably, psychiatric drugs as a class are over represented. Antipsychotics such as chlorpromazine, phenothiazine, and clozapine are known culprits as well are the antidepressants such as trazodone [5]. Olanzapine or methylphenidate therapy (drugs used for attention deficit hyperactivity disorder) is reported to cause priapism in children [6]. Total parenteral nutrition, with its high fat content, is also believed to cause priapism [7]. Hematological diseases that are accompanied by hyperviscosity such as sickle cell disease or trait and leukemia are well-known causes of ischemic priapism in children [8].

High-flow (nonischemic) priapism was firstly described over 40 years ago [9]. It is always the result of a straddle injury of the corpora cavernosal resulting in an arteriocavernosal fistula, which on its turn results in an uncontrolled arterial inflow into the cavernosal sinusoids. Because the cavernosal smooth muscle is contracted and well oxygenated (on aspiration, bright red blood with a high PO2 is encountered), the erection is semirigid and not painful [8]. Gold standard in the diagnosis of high-flow priapism is the demonstration of turbulent blood flow through the arteriocavernosal fistula with the aid of perineal colour Doppler flowmetry [10, 11]. The treatment of choice is highly selective embolisation of the affected cavernosal artery with gel foam or an autologous blood clot [8].

In low-flow priapism, venous outflow is blocked due to a complete paralysis of the cavernous smooth muscle. Due to ischemia, the erection is painful. One aspiration dark blood (with an acidic PH and low P02) is found. Primary treatment consists of cavernosal aspiration and corporal rinsing with norepinephrine or methylene blue. When the priapism appears to be resistant to this first line treatment, a surgical shunting procedure designed to improve penile venous drainage can be performed. This will only be successful if a frozen section of a cavernosal biopsy has shown the existence of vital cavernosal smooth muscle tissue. In case of necrosis, a shunting procedure is likely to fail, and implantation of an erection prosthesis to prevent penile future fibrosis and shortening may be warranted (personal communication). Thus, the corporeal frozen section could be an option for clinical decision-making and opposed to the standard shunting then awaiting clinical result before placement of prosthesis.

Various histological studies have been performed to define the histological changes in relation with the duration of ischemia. In the early stages at less than 12 hours, interstitial edema and thickening are the most common findings. From 12 to 24 hours, thrombocytes start to adhere to the endothelium such that by 48 hours necrosis of cavernosal smooth muscle cells and fibroblast proliferation has occurred. The end result is loss of smooth muscle and fibrosis of the corpora cavernosa [12].

Low-flow priapism has been described in men of all ages including newborns. The peak incidence is bimodal with the highest incidence occurring between the ages of 5–10, and 20–50 years. Generally in the younger age group, priapism is secondary to a neoplasm or sickle cell anemia [8].

The main cause of priapism in children is haematological disorders: most notably sickle cell disease or acute leukaemia. In these conditions, priapism may be associated with general signs and symptoms of, for example, a sickle cell crisis involving other areas of the body. However, very often it presents as an isolated event [13]. Initial treatment should be aimed at the systemic management of the underlying condition (e.g., in sickle cell disease, rapid hydration exists with IV fluids between two to three times in the maintenance rate) and analgesia with meperidine or morphine, and hyper transfusion with packed red blood cells to increase the hemoglobin to above 10 to 12 mg per dl [14]. Furthermore, a trial of corporal aspiration and instillation of norepinephrine should be attempted. Fortunately, most cases of sickle cell priapism in children resolve promptly with these measures. However, if this fails and the penis remains erect and painful longer then 6 hours, more aggressive surgical management with glandular corporal or spongiocorporal shunt may be required [15]. This can be effective up to 24–48 hours after the onset of the priapism, depending on the result of the frozen section of cavernous smooth muscle tissue [10]. Should priapism recur after the initial episode? The condition is called stuttering or recurrent priapism [16–18]. The pathophysiology of stuttering priapism is unknown. It is speculated that downregulation of adrenoreceptors in the cavernous smooth musculature or scarring of intracavernous venules may trigger the recurrences [8]. In the literature, several empirical treatments are advocated to prevent recurrences, such as low-dose aspirin and digitalis. Recently, Burnett et al. postulated that glucose-6-phosphate dehydrogenase deficiency (G6PD deficiency) may offer an explanation for idiopathic priapism. They observed that priapism recurrences were sufficiently resolved with low-dose PDE5 inhibitor therapy and explain this observation by the fact that G6PD deficiency generates a pathophysiologic milieu consistent with aberrant nitric oxide (NO)-signalling and heightened oxidative stress in the corpus cavernosum [19].

In general, treatment is aimed at the primary cause of the priapism if it can be identified. The ultimate goal of treatment is to relieve the pain and stop the erection to prevent damage to the corporal bodies.

## 4. CONCLUSION

In prepubertal boys, idiopathic recurrent priapism is a rare condition. In the literature, several empirical therapies are described. Recently, it is postulated that glucose-6-phosphate dehydrogenase deficiency may offer an explanation for the pathophysiological mechanism and that the condition may be resolved with a low dose of a PDE5 inhibitor.
